# Inflammatory Markers and the Risk of Chronic Obstructive Pulmonary Disease: A Systematic Review and Meta-Analysis

**DOI:** 10.1371/journal.pone.0150586

**Published:** 2016-04-22

**Authors:** Bin Su, Tiansheng Liu, Haojun Fan, Feng Chen, Hui Ding, Zhouwei Wu, Hongwu Wang, Shike Hou

**Affiliations:** 1 Medical Simulation Teaching Base, Affiliated Hospital of Logistics University of Chinese People’s Armed Police Forces, Tianjin, China; 2 Department of Orthopedics, Affiliated Hospital of Logistics University of Chinese People’s Armed Police Forces, Tianjin, China; 3 Affiliated Hospital of Logistics University of Chinese People’s Armed Police Forces, Tianjin, China; 4 Tianjin University of Traditional Chinese Medicine, Tianjin, China; University of Texas Health Science Center at Tyler, UNITED STATES

## Abstract

Systemic inflammatory factors are inconsistently associated with the pathogenesis of chronic obstructive pulmonary disease (COPD). We conducted a systematic review and meta-analysis to summarize the evidence supporting the association between systemic inflammation and the risk of COPD. Pertinent studies were retrieved from PubMed, EmBase, and the Cochrane Library until April 2015. A random-effects model was used to process the data, and the analysis was further stratified by factors affecting these associations. Sensitivity analyses for publication bias were performed. We included 24 observational studies reporting data on 10,677 COPD patients and 28,660 controls. Overall, we noted that COPD was associated with elevated serum CRP (SMD: 1.21; 95%CI: 0.92–1.50; P < 0.001), leukocytes (SMD: 1.07; 95%: 0.25–1.88; P = 0.010), IL-6 (SMD: 0.90; 95%CI: 0.48–1.31; P < 0.001), IL-8 (SMD: 2.34; 95%CI: 0.69–4.00; P = 0.006), and fibrinogen levels (SMD: 0.87; 95%CI: 0.44–1.31; P < 0.001) when compared with control. However, COPD was not significantly associated with TNF-α levels when compared with control (SMD: 0.60; 95%CI: -0.46 to 1.67; P = 0.266). Our findings suggested that COPD was associated with elevated serum CRP, leukocytes, IL-6, IL-8, and fibrinogen, without any significant relationship with TNF-α.

## Introduction

Chronic obstructive pulmonary disease (COPD) is one of the major causes of death [1]. COPD is characterized by progressive and incompletely reversible airway obstruction. It is associated with an abnormal systemic inflammatory response of the lungs to particles or noxious gases [2–3]. The mechanisms associated with the risk of COPD are inconclusive, and are most likely related to systemic inflammation, oxidative stress, hypoxia and a sedentary lifestyle [4,5]. Previous studies already demonstrated that cigarette smoking is the major risk factor for the development and progression of COPD [6–8]. Although many biomarkers play an important role in the pathophysiology of COPD, their role in monitoring COPD outcomes is unclear.

Several retrospective studies have suggested that active inflammation marked by increased serum levels of tumor necrosis factor-α (TNF-α), interleukin (IL-6), and C-reactive protein (CRP) was associated with the progression of COPD [9–15]. However, the results of 4 studies [14,16–18] showed no association between CRP and the risk of COPD. The study by Harting et al [18] suggested that patients with COPD had lower serum IL-6 levels, and five studies suggested that COPD was associated with lower or insignificant serum TNF-α levels [19–23]. Further, the association between IL-8, leukocyte, or fibrinogen levels and the risk of COPD remains inconsistent.

In 2004, Gan et al [24] conducted a systematic review and meta-analysis of 14 studies, which suggested that increased levels of systemic inflammatory markers were associated with lower lung function. However, few studies investigated the role of specific markers in COPD. Therefore, we attempted to conduct a comprehensive meta-analysis of the available studies to determine the association between markers of serum inflammation and the risk of COPD. We also compared these associations in participants with different smoking status.

## Materials and Methods

### Data sources, search strategy, and selection criteria

Our study was conducted and reported according to the Preferred Reporting Items for Systematic Reviews and Meta-Analysis Statement issued in 2009 (S1 Checklist) [25]. We systematically searched PubMed, EmBase, and the Cochrane Library for articles published through April 2015, to identify potential observational studies, which evaluated the association between systemic inflammatory markers and COPD pathogenesis. We used a search strategy combining text and MeSH terms as follows: (“COPD” OR “chronic obstructive pulmonary disease” OR “bronchitis” OR “emphysema” OR “forced expiratory volume” OR “vital capacity”) AND (“systemic inflammation” OR “biological markers” OR “C-reactive protein” OR “CRP” OR “fibrinogen” OR “leucocyte” OR “interleukin” OR “interleukin 6” OR “interleukin-6” OR “IL-6” OR “interleukin 8” OR “interleukin-8” OR “IL-8” OR “tumor necrosis factor-α” OR “TNF-α” OR “inflammatory cytokines”). We restricted any potential studies that were published in English. Abstracts and online presentations were also searched to identify relevant unpublished clinical studies. Finally, we hand-searched articles with published data relevant to our search as well as the reference lists of all retrieved articles and relevant reviews, comments, or letters. Medical subject headings, methods, disease status, design, and levels of systemic inflammatory markers were used to identify the relevant studies.

The literature search was undertaken by two authors independently. Any inconsistencies between the two authors were resolved by group discussion to arrive at a consensus. The inclusion criteria of studies were: (1) patients with COPD; (2) participants in control group without COPD; (3) studies reporting at least one of the following markers: CRP, IL-6, IL-8, TNF-α, leukocyte, or fibrinogen levels in COPD and control groups; and (4) studies reporting mean, standard deviation and sample size for comparison of case and control for each inflammatory factor. In case of studies without adequate data, we contacted the authors or searched similar articles, which reported sample populations to obtain the unpublished results.

### Data collection and quality assessment

Data extraction and assessment were performed independently by two reviewers. Publication information (first author’s name, publication year), characteristics of patients and control (country, sample size, mean age, gender, percentage of smoker; patients’ status, control status, and assessment of exposure), and outcome variables (CRP, IL-6, IL-8, TNF-α, leukocyte, or fibrinogen levels) were extracted. Any disagreement was resolved by consensus with a third reviewer.

Two reviewers independently evaluated the quality of the included study using the Newcastle-Ottawa Scale (NOS) [26], which was based on the following 3 subscales: selection (4 items), comparability (1 item), and outcome (3 items). A “star system” (range, 0–9) was developed for assessment. In case of disagreement, a consensus was reached after group discussion.

### Statistical analysis

We examined the relationship between markers of systemic inflammation and COPD pathogenesis on the basis of the mean, standard deviation, and sample size in each group, published in each study. Standard mean difference (SMD) was used as a summary statistic. The SMD was significant when the 95% confidence interval (CI) did not include 0. We pooled the SMDs for each inflammatory marker and COPD pathogenesis using random-effects models [27,28]. Heterogeneity between studies was investigated using the Q statistic, and we considered P values < 0.10 as indicative of significant heterogeneity [29,30]. Subgroup analyses were conducted for the incidence of COPD on the basis of country, mean age, current smoking status, and FEV_1_ predicted. Sensitivity analyses were also conducted by removing each individual study from the meta-analysis [31]. Egger [32] and Begg [33] tests were also used to quantify publication bias. All reported P values are 2-sided, and P values < 0.05 were considered statistically significant. Data analyses were performed using STATA software (version 12.0; Stata Corporation, College Station, TX, USA).

## Results

**Fig 1:**
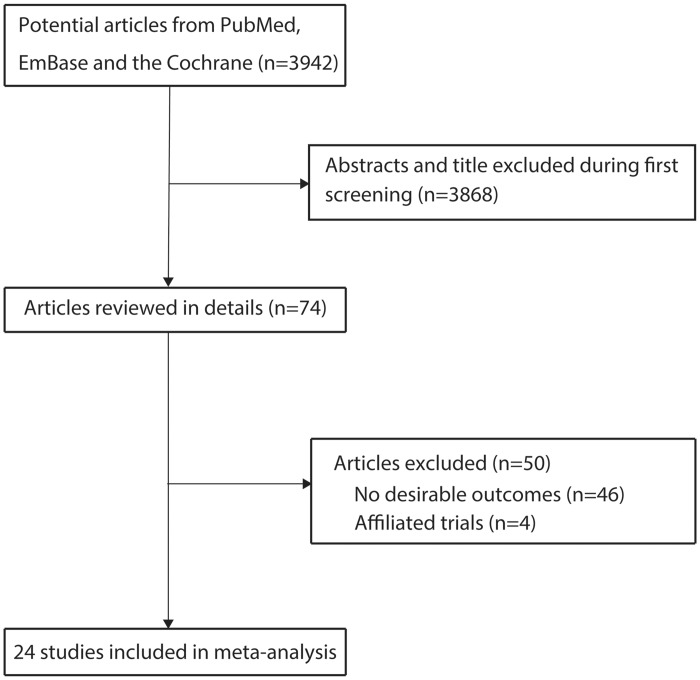
Flow diagram outlining the literature search strategy and study selection process.

The primary search produced 3,942 records. After scanning titles and abstracts, we excluded 3,868 irrelevant or duplicate articles. A total of 74 potentially eligible studies were selected, and after detailed evaluation, 24 observational studies [9–23,34–42] were selected for the final pooled analysis (Fig 1). A manual search of the reference lists of these studies did not yield any new eligible studies. Table 1 presents the general characteristics of the included studies. In these 24 studies including a total of 10,677 COPD cases, and 28,660 controls, 22 were case control studies and the remaining 2 were cross sectional studies. Five studies [14,18,37,38,40] were conducted in America, 12 in Europe [9,13,15,16,19–21,23,34,36,41,42], 5 in Asia [10–12,35,39], 1 in Africa [17], and the remaining 1 in multiple countries [22]. The mean age of COPD cases was 52.2–73.1 years, while it was 49.0–74.5 years in the control group in each included study. Further, the percentage of smokers ranged from 0 to 60.9% in each study. Study quality was assessed using the NOS. Notably, all the included case control studies scored at least 6/9 on the NOS.

**Table 1:** Baseline characteristics of included studies.

Study	Country	Sample size	Mean age	Gender (M/F)	Current smoker (%)	COPD patient status	Control status	Reported outcomes
SD Aaron [14]	Canada	21/21	68.8/62.3	17/25	40.5	The mean post-bronchodilator FEV1 was 46% and 44% of the predicted value in non-smoking and smoking cohorts with COPD, respectively	Lung function was normal	CRP↑, IL-6↑, IL-8↓
MP FoschinoBarbaro [9]	Italy	27/15	52.0/49.0	33/9	0	Basal FEV_1_: 1.9 and 75.3% of predicted value	Basal FEV_1_: 2.6 and 110.4% of predicted value	CRP↑, IL-6↑, TNF-α↑
A Bircan [34]	Turkey	30/30	66.6/66.7	60/0	0	Basal FEV_1_: 1.22 and 44.7% of predicted value	Basal FEV_1_: 3.25 and 90.3% of predicted value	CRP↑
S Hacker [15]	Austria	35/29	59.6/56.9	37/27	60.9	FEV1 was 70.21% and 30.67% of the predicted value in COPD stages I or II and COPD III or IV, respectively	FEV1 was94.40% and 105.37% of the predicted value in smokers and non-smokers	IL-6↑, CRP↑
Z He [35]	China	44/20	60.1/55.5	57/7	NR	FEV1 was 95.5%, 58.7%, 35.1% and 25.3% of the predicted levels in stages I, II, III, and IV COPD, respectively.	FEV1 was 108.3% and 107.6% of the predicted value in smokers and non-smokers	Leukocytes↑, IL-6↑, CRP↑
Van Helvoort HA [19]	Netherlands	20/10	65.5/59.0	19/11	0	Basal FEV_1_: 1.35 and 53.5% of predicted level	Basal FEV_1_: 3.3 and 108% of predicted level	CRP↑, leukocyte↑, IL-6↑, IL-8↑, TNF-α↓
F Karadag [20]	Turkey	35/30	65.6/63.2	65/0	0	FEV_1_: 51.36% of predicted level	FEV_1_: 85.09% of predicted value	CRP↑, TNF-α↑, IL-6↑
AL Kersul [36]	Spain	17/28	66.6/62.1	35/10	37.8	Basal FEV_1_: 1.39 and 41.7% of predicted value	Basal FEV_1_: 2.92 and 2.45 in smokers and non-smokers, respectively; 98.5% and 117.5 of predicted level in smokers and non-smokers, respectively	CRP↑, leucocytes↑, fibrinogen↑
X Liu [10]	China	35/28	70.0/70.0	63/0	0	FEV_1_: 59.5% of predicted value	FEV_1_: 75.1% of predicted value	CRP↑, TNF-α↑
DM Mannino [37]	US	2366/12791	NR	7384/8313	30.0	Mild, moderate and severe COPD	Respiratory symptoms only, Restrictive lung disease, and no lung disease	CRP↑, Fibrinogen↑
AMW Petersen [21]	Denmark	19/20	66.0/64.0	16/23	5.1	FEV_1_: 31.0% of predicted value	FEV_1_: 96.0% of predicted value	CRP↑, IL-6↑, TNF-α↓
VM Pinto-Plata [38]	US	88/71	66.0/64.7	97/62	20.8	FEV_1_: 37.0% of predicted value	FEV1 was91.0% and 92.0% of the predicted level in smokers and non-smokers, respectively	CRP↑
A Undas [16]	Poland	56/56	64.9/63.8	99/13	37.9	FEV_1_: 54.7% of predicted level	FEV_1_: 92.2% of predicted level	CRP↑, fibrinogen↑
N Yasuda [11]	Japan	39/42	66.0/66.4	56/25	32.1	VC: 81.9% and 70.6 of predicted value in mild or moderate COPD and severe COPD, respectively	VC: 46.2% and 92.6% of predicted value in disease control and health control, respectively	CRP↑, IL-6↑, TNF-α↑
M Fattouh [17]	Egypt	98/30	62.29/NR	105/23	22.7	FEV_1_: 58.58% of predicted value	FEV_1_: 88.4% of predicted value	CRP↓,leukocytes↓,fibrinogen↑
E Feng [39]	China	62/40	57.9/58.1	61/41	NR	FEV_1_: 32.34% of predicted value	FEV_1_: 71.15% of predicted value	IL-8↑
X Wang [12]	China	58/29	73.1/74.5	63/24	59.8	FEV_1_: 48.3% of predicted value	FEV_1_: 71.5% of predicted value	CRP↑, IL-6↑, IL-8↑, TNF-α↑
JR Harting [18]	US	10/10	62.2/53.2	16/4	50.0	FEV_1_: 51.0% of predicted value	FEV_1_: 96.0% of predicted value	CRP↑, leukocyte↓, IL-6↓, IL-8↑
A Agusti [22]	Multi-center	1755/499	63.5/54.5	1398/856	36.7	Basal FEV_1_: 1.35 and 48.2% of predicted value	Basal FEV_1_: 3.31 and 3.34 in smokers and non-smokers, respectively; 108.8% and 115.3% of predicted value in smokers and non-smokers, respectively	CRP↑, leukocyte↑, IL-6↑, IL-8↑, fibrinogen↑, TNF-α—
D Valvi [40]	US	5475/14717	NR	9011/11181	24.4	NR	NR	Fibrinogen↑
B van den Borst [41]	Netherlands	28/15	65.0/65.0	26/17	25.6	FEV_1_: 58.0% of predicted value	FEV_1_: 113.0% of predicted value	CRP↑, IL-6↑
F Garcia-Rio [13]	Spain	324/110	64.0/55.0	292/142	30.6	Basal FEV_1_: 2.03 and 77.0% of predicted value	Basal FEV_1_: 3.13 and 115.0% of predicted value	CRP↑, IL-8↑, TNF-α↑
E Barreiro [23]	Spain	19/7	64.0/63.0	26/0	NR	FEV_1_: 33.0% of predicted value	FEV_1_: 110.0% of predicted value	IL-6↑, IL-8↑, TNF-α↓
E Bucchioni [42]	Italy	16/12	63.9/57.3	18/10	NR	Basal FEV_1_: 1.2 and 41.1% of predicted level	Basal FEV_1_: 2.9 and 93.3% of predicted level	IL-6↑

Symbols means: “↑”: enhancement; “↓”: inhibition; “—”: invariant.

**Fig 2:**
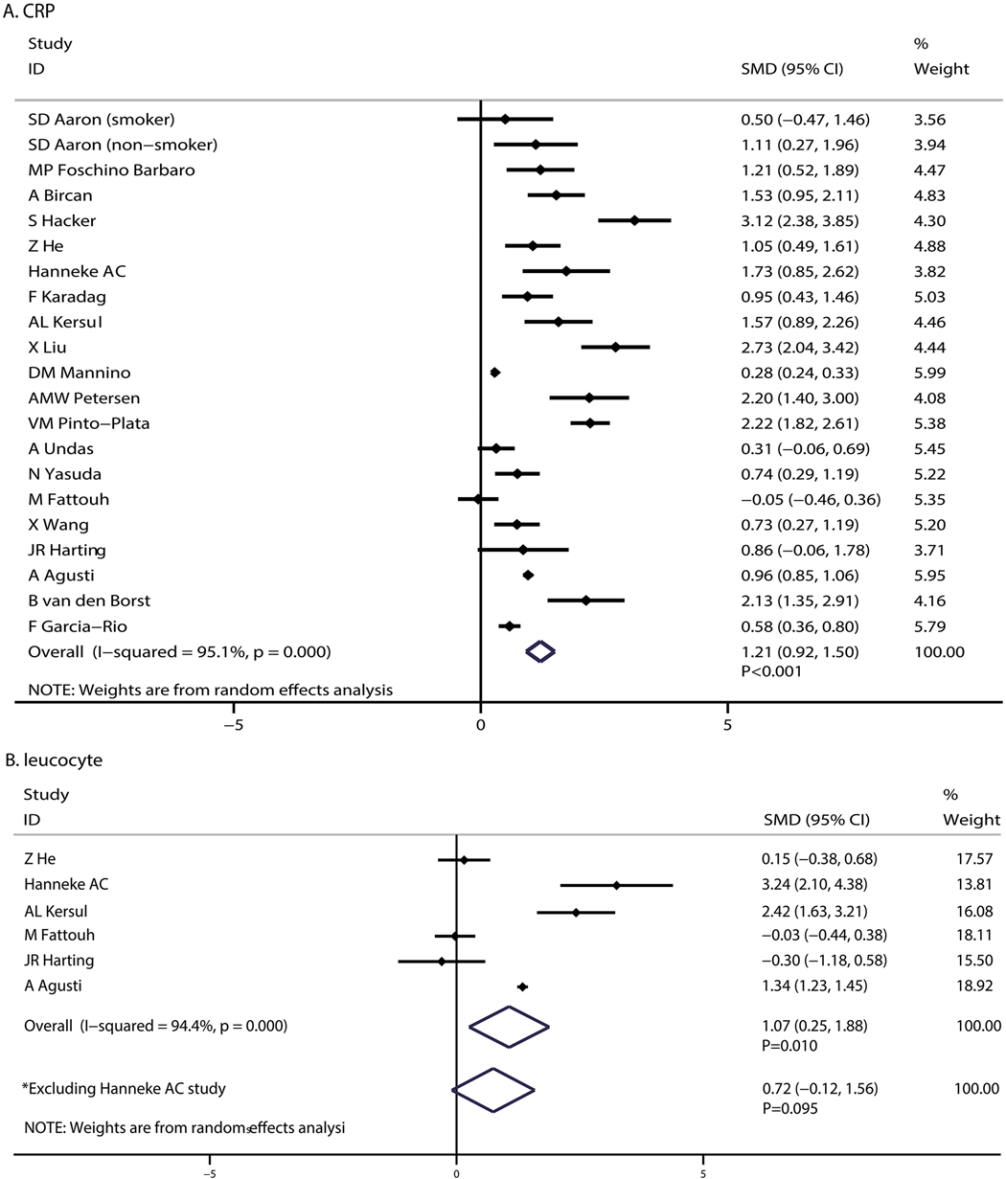
COPD associated with CRP (A) and leukocyte levels (B).

A total of 21 cohorts in 20 studies reported an association between CRP and the pathogenesis of COPD. The pooled SMD showed that COPD was associated with higher CRP levels when compared with control (SMD: 1.21; 95%CI: 0.92–1.50; P < 0.001; Fig 2A), and substantial heterogeneity was presented (I-square: 95.1%; P < 0.001). As a result, a sensitivity analysis was conducted, and after each study was sequentially excluded from the pooled analysis, the conclusion was not affected by the exclusion of any specific study.

A total of 6 studies reported the relationship between leukocyte levels and COPD pathogenesis. We noted that patients with COPD presented with high leukocyte levels when compared with participants without COPD (SMD: 1.07; 95%: 0.25–1.88; P = 0.010; Fig 2B), although significant heterogeneity was detected (I-square: 94.4%; P < 0.001). Based on the sensitivity analysis, we excluded the study conducted by Helvoort et al [19], which specifically separated COPD patients into muscle wasted and non-muscle wasted categories, which may have contributed significantly to this association. Subsequently, we concluded that COPD was not associated with leukocyte levels (SMD: 0.72; 95%CI: -0.12 to 1.56; P = 0.095; with substantial heterogeneity: I-square = 94.8%; P < 0.001; Fig 2B).

**Fig 3:**
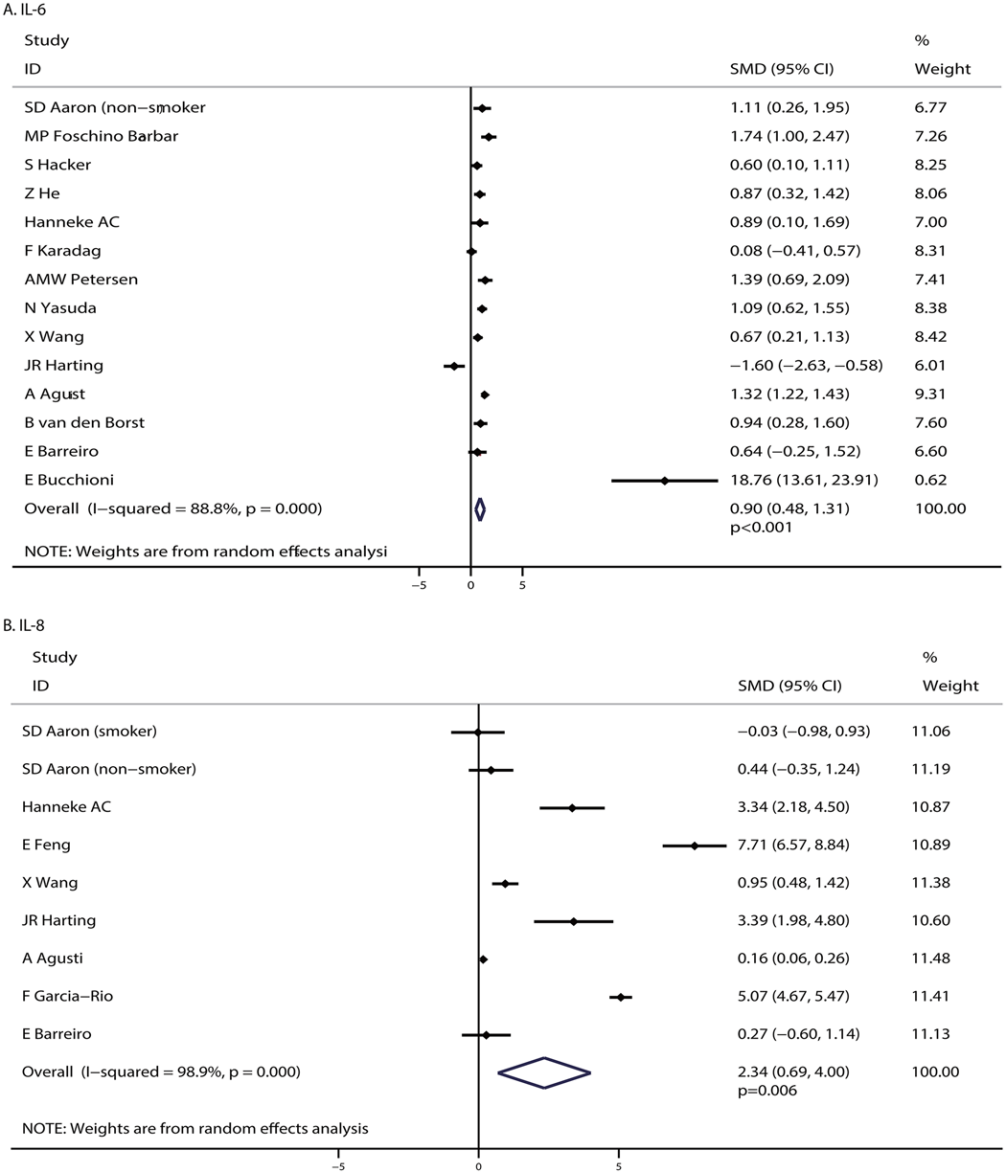
COPD associated with IL-6 (A) and IL-8 levels (B).

The breakdown of the number of studies available for each outcome was 14 cohorts (14 studies) and 9 cohorts (8 studies) for IL-6 and IL-8 levels, respectively. The pooled analysis of the relationship between IL-6 and IL-8 levels with COPD pathogenesis suggested that COPD patients showed higher IL-6 (SMD: 0.90; 95%CI: 0.48–1.31; P < 0.001; Fig 3A) and IL-8 levels (SMD: 2.34; 95%CI: 0.69–4.00; P = 0.006; Fig 3B) compared with the controls. We observed substantial heterogeneity in the magnitude of the effect across the studies (P < 0.001 for IL-6 and IL-8).We conducted sensitivity analyses and the conclusion was not altered after sequential exclusion of each study from all the pooled analyses.

**Fig 4:**
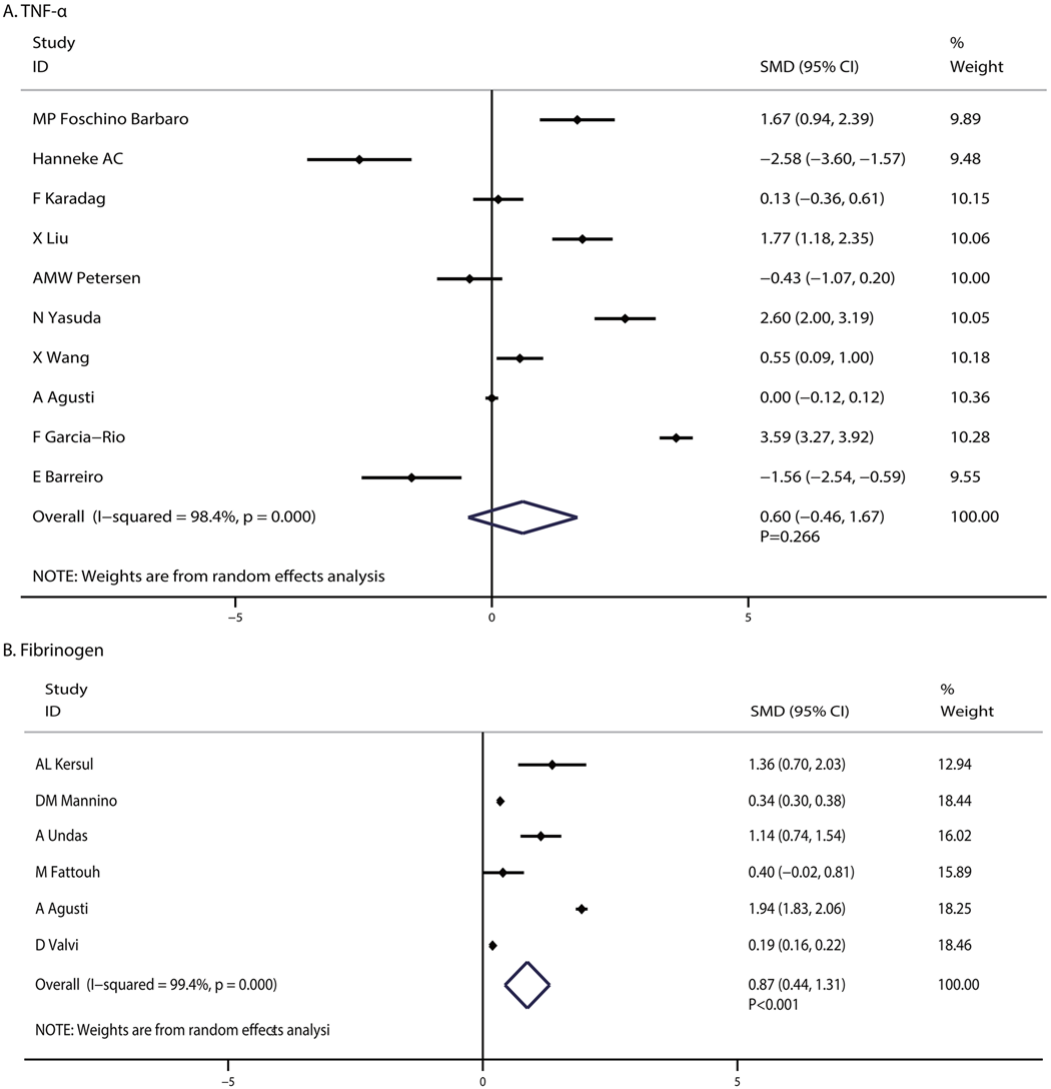
COPD associated with TNF-α (A) and fibrinogen levels (B).

A total of 10 articles reported an association between TNF-α level and the risk of COPD. The pooled SMD showed no significant difference between COPD patients and control (SMD: 0.60; 95%CI: -0.46 to 1.67; P = 0.266; Fig 4A), and substantial heterogeneity was observed (I-square: 98.4%; P < 0.001). A sensitivity analysis suggested that the conclusion was not affected by the exclusion of any specific study.

A total of 6 studies reported the relationship between fibrinogen level and the pathogenesis of COPD. We found that COPD was associated with elevated fibrinogen levels when compared with participants without COPD (SMD: 0.87; 95%CI: 0.44–1.31; P < 0.001; Fig 4B). Although substantial heterogeneity was observed in the magnitude of the effect across the studies (I-square: 99.4%; P < 0.001), the conclusion was not affected by the exclusion of any specific study.

Heterogeneity testing of the analysis showed a value of P < 0.001 for systemic inflammatory markers. Therefore, we conducted subgroup analyses to minimize heterogeneity among the included studies (Table 2). Overall, COPD was not related to leukocyte levels if the study was conducted in other countries, the mean age of patients was less than 60, the participants were current or partial smokers, and FEV_1_ predicted in COPD patients was greater than 50%. Similarly, COPD patients were not associated with IL-6 level if the participants were non-smokers. Patients with COPD were not associated with elevated IL-8 level if the study was conducted in Europe, or participants were current or partial smokers. Finally, COPD was associated with elevated TNF-α level if the study was conducted in other countries, and the mean age of participants was less than 60.

**Table 2:** Subgroup analysis of SMD: COPD patients vs. control.

Variables	Group	SMD and 95%CI	P value	Heterogeneity (%)	P value for heterogeneity
CRP	Country				
	Europe	1.28 (0.85, 1.72)	< 0.001	83.8	< 0.001
	Other	1.15 (0.76, 1.55)	< 0.001	96.7	< 0.001
	Mean age				
	> 60	1.18 (0.88, 1.48)	< 0.001	88.6	< 0.001
	< 60	1.78 (0.54, 3.01)	0.005	90.5	< 0.001
	Current smoking status				
	Yes or partial	1.09 (0.77, 1.41)	< 0.001	95.8	< 0.001
	no	1.61 (1.00, 2.22)	< 0.001	77.1	0.002
	FEV_1_ predicted				
	> 50%	1.25 (0.74, 1.76)	< 0.001	91.1	< 0.001
	< 50%	1.33 (0.93, 1.73)	< 0.001	84.6	< 0.001
Leukocytes	Country				
	Europe	2.73 (1.95, 3.50)	< 0.001	25.8	0.246
	Other	0.33 (-0.61, 1.26)	0.493	95.5	< 0.001
	Mean age				
	> 60	1.27 (0.33, 2.20)	0.008	94.5	< 0.001
	< 60	0.15 (-0.38, 0.68)	0.569	.	.
	Current smoking status				
	Yes or partial	0.86 (-0.12, 1.84)	0.086	95.0	< 0.001
	no	3.24 (2.10, 4.38)	< 0.001	.	.
	FEV_1_ predicted				
	> 50%	0.91 (-0.84, 2.65)	0.308	93.3	< 0.001
	< 50%	1.26 (0.31, 2.22)	0.009	92.4	< 0.001
IL-6	Country				
	Europe	1.48 (0.50, 2.46)	0.003	90.6	< 0.001
	Other	0.70 (0.23, 1.17)	0.003	87.0	< 0.001
	Mean age				
	> 60	0.87 (0.34, 1.40)	0.001	90.7	< 0.001
	< 60	1.02 (0.42, 1.62)	0.001	68.0	0.044
	Current smoking status				
	Yes or partial	0.79 (0.37, 1.22)	< 0.001	84.6	< 0.001
	no	0.87 (-0.14, 1.87)	0.092	85.7	0.001
	FEV_1_ predicted				
	> 50%	0.59 (0.00, 1.17)	0.048	83.7	< 0.001
	< 50%	1.26 (0.62, 1.90)	< 0.001	89.3	< 0.001
IL-8	Country				
	Europe	2.91 (-0.24, 6.05)	0.070	98.0	< 0.001
	Other	2.02 (0.57, 3.47)	0.006	97.4	< 0.001
	Mean age				
	> 60	1.69 (0.08, 3.29)	0.039	98.8	< 0.001
	< 60	7.71 (6.57, 8.84)	< 0.001	.	.
	Current smoking status				
	Yes or partial	1.65 (-0.28, 3.59)	0.093	99.1	< 0.001
	no	3.34 (2.18, 4.50)	< 0.001	.	.
	FEV_1_ predicted				
	> 50%	4.04 (2.69, 5.40)	< 0.001	82.9	0.003
	< 50%	1.51 (0.20, 2.83)	0.024	97.2	< 0.001
TNF-α	Country				
	Europe	0.16 (-1.81, 2.13)	0.874	98.5	< 0.001
	Other	1.20 (0.04, 2.36)	0.042	97.1	< 0.001
	Mean age				
	> 60	0.49 (-0.66, 1.63)	0.404	98.5	< 0.001
	< 60	1.67 (0.94, 2.39)	< 0.001	.	.
	Current smoking status				
	Yes or partial	1.26 (-0.42, 2.95)	0.142	99.2	< 0.001
	no	0.29 (-1.25, 1.83)	0.716	95.4	< 0.001
	FEV_1_ predicted				
	> 50%	1.23 (-0.31, 2.76)	0.117	97.8	< 0.001
	< 50%	-0.21 (-0.77, 0.35)	0.460	82.7	0.001
Fibrinogen	Country				
	Europe	1.20 (0.86, 1.54)	< 0.001	0.0	0.575
	Other	0.73 (0.21, 1.24)	0.006	99.6	< 0.001
	Mean age				
	> 60	1.22 (0.43, 2.01)	0.003	95.2	< 0.001
	< 60	.	.	.	.
	Current smoking status				
	Yes or partial	0.87 (0.44, 1.31)	< 0.001	99.4	< 0.001
	no	.	.	.	.
	FEV_1_ predicted				
	> 50%	0.77 (0.04, 1.50)	0.039	84.6	0.011
	< 50%	1.75 (1.22, 2.28)	< 0.001	64.2	0.095

The Egger [32] and Begg tests [33] showed no evidence of publication bias for CRP (P value for Egger: 0.578; P value for Begg: 0.144), leukocytes (P value for Egger: 0.395; P value for Begg: 0.308), IL-8 (P value for Egger: 0.201; P value for Begg: 0.386), TNF-α (P value for Egger: 0.688; P value for Begg: 0.707), and fibrinogen (P value for Egger: 0.339; P value for Begg: 0.452). However, although the Begg test showed no evidence of publication bias for IL-6 (P = 0.669), the Egger test showed potential evidence of publication bias for IL-6 (P = 0.001). The conclusions were not altered after adjusting for publication bias using the “trim and fill” method.

## Discussion

The objective of the present meta-analysis was to determine the relationship between systemic inflammatory markers and COPD pathogenesis. Twenty-four observational studies were identified (22 case control studies, and 2 cross-sectional studies), and included a total of 10,677 COPD cases, and 28,660 controls. Results from pooled analyses showed that COPD was associated with an elevated CRP, leukocyte, IL-6, IL-8, and fibrinogen levels when compared with control group, without any significant differences in TNF-α levels. These results enable us to refine the definition of clinical phenotypes and monitor the response to existing and new therapeutic strategies, and assist physicians with accurate therapeutic decision-making.

A previous meta-analysis [24] suggested that reduced lung function was associated with increased levels of systemic inflammatory markers, which may have important pathophysiological and therapeutic implications for subjects with stable COPD. The inherent limitation of the previous review related to the small number of studies reporting several markers. For instance, only 1 study reported the relationship between IL-6 and COPD pathogenesis. Two studies provided the outcomes with IL-8, which were not statistically significant, and the outcomes were unstable. Therefore, we conducted an updated meta-analysis to determine these associations, and evaluate the relationships in specific subpopulations.

Most of our findings were consistent with previous studies. However, several studies reported inconsistent results. Aaron et al [14] suggested the absence of significant differences between CRP and pathogenesis of COPD in smokers. Further, Fattouh et al [17] indicated that elevated levels of CRP, fibrinogen and leukocytes in individuals with COPD were associated with increased risk of exacerbation, but no significant difference when COPD patients were compared with controls. The result could be explained by the study design exploring systemic inflammatory markers in patients with different COPD status, and the different stages of COPD across included studies. Our current study suggested that CRP and leukocyte count played an important role in COPD pathogenesis. CRP and leukocyte levels may be markers of significant bacterial infection providing a rationale for antibiotic treatment.

We observed that COPD was associated with elevated IL-6, and IL-8 levels when compared with control. However, several studies included in our study reported inconsistent results. Harting et al [18] suggested that COPD patients contained lower levels of IL-6. Furthermore, Aaron et al [14] indicated that IL-8 level was not associated with COPD pathogenesis. The inconsistent findings may be related to the inclusion of studies with different baseline characteristics and early stages of COPD that are insensitive to systemic inflammatory markers.

Previous study suggested that fibrinogen was probably a useful biomarker to stratify individuals with a high or low risk of COPD exacerbation [40]. In our study, individuals with COPD were associated with elevated fibrinogen levels. Fibrinogen might represent a potentially useful biomarker and requires large observational studies for further validation.

No significant association was seen between TNF-α level and the pathogenesis of COPD. In our pooled analysis of 10 studies, five reported that COPD was associated with elevated TNF-α level, whereas two studies found COPD associated with lower TNF-α level, and the remaining 3 reported no significant relationship. A previous meta-analysis [24] also suggested that COPD was associated with higher TNF-α level with only 4 studies reporting this outcome. In comparison, in our current meta-analysis, 10 of the included studies reported this relationship. In addition, a few included studies [9, 20, 10, 11, 22] definitely indicated that TNF-α was a mediator of cachexia and inflammation induced apoptosis. According to Garcia-Rio et al [13], the origin of systemic inflammation in COPD and the association between TNF-α level and the pathogenesis of COPD were not completely clear. Similarly, substantial heterogeneity and variance observed in the included studies preclude any unequivocal conclusion. Our comparative study provides a comprehensive analytical review.

The strengths of our study relate to the large sample size allowing us to quantitatively evaluate the relationship of systemic inflammation with the pathogenesis of COPD. Therefore, our findings are potentially more robust than those of any individual study. Further, we summarized six important markers and their potential role in COPD, and explored the relationships in several specific subpopulations.

The limitations of our meta-analysis relate to the use of pooled data. Individual data were not available, which precluded a more detailed and comprehensive analysis. In addition, meta-analyses always use published studies, and publication bias is inevitable.

## Conclusion

In conclusion, the results of our study indicate that systemic inflammatory markers play an important role in the pathogenesis of COPD. Further large-scale studies are needed to corroborate the findings, before providing recommendations for identifying clinical phenotypes and monitoring therapeutic response.

## Supporting Information

S1 Checklist PRISMA 2009 Checklist. (DOC)
